# Early Detection of Preeclampsia Using Circulating Small non-coding RNA

**DOI:** 10.1038/s41598-018-21604-6

**Published:** 2018-02-21

**Authors:** Liron Yoffe, Avital Gilam, Orly Yaron, Avital Polsky, Luba Farberov, Argyro Syngelaki, Kypros Nicolaides, Moshe Hod, Noam Shomron

**Affiliations:** 1 Faculty of Medicine, Tel Aviv University, Tel Aviv, Israel; 2 Harris Birthright Research Centre for Fetal Medicine, King’s College, London, UK; 3 Rabin Medical Center, Petah-Tikva, Israel

## Abstract

Preeclampsia is one of the most dangerous pregnancy complications, and the leading cause of maternal and perinatal mortality and morbidity. Although the clinical symptoms appear late, its origin is early, and hence detection is feasible already at the first trimester. In the current study, we investigated the abundance of circulating small non-coding RNAs in the plasma of pregnant women in their first trimester, seeking transcripts that best separate the preeclampsia samples from those of healthy pregnant women. To this end, we performed small non-coding RNAs sequencing of 75 preeclampsia and control samples, and identified 25 transcripts that were differentially expressed between preeclampsia and the control groups. Furthermore, we utilized those transcripts and created a pipeline for a supervised classification of preeclampsia. Our pipeline generates a logistic regression model using a 5-fold cross validation on numerous random partitions into training and blind test sets. Using this classification procedure, we achieved an average AUC value of 0.86. These findings suggest the predictive value of circulating small non-coding RNA in the first trimester, warranting further examination, and lay the foundation for producing a novel early non-invasive diagnostic tool for preeclampsia, which could reduce the life-threatening risk for both the mother and fetus.

## Introduction

Preeclampsia (PE) is one of the most dangerous pregnancy complications, affecting 3–8% of pregnancies; it is the leading cause of maternal and perinatal mortality and morbidity^1,2^. It occurs in the second or third trimester, and is characterized by de-novo development of concurrent hypertension with either proteinuria or at least one severe feature (thrombocytopenia, renal insufficiency, impaired liver function, pulmonary edema, cerebral or visual symptoms) after gestational week 20^3^. PE is called “the disease of theories” reflecting the lack of understanding of its pathogenesis. Several pathophysiological mechanisms have been proposed in the development of PE, including endothelial dysfunction^4^, an inflammatory pathway^5^, oxidative stress^6^, and angiogenic factors disregulation^7^; though it still remains poorly understood^8^. It is commonly accepted that the placenta has a central role in the pathogenesis of PE, and it is assumed that PE originates from poor placentation, which involves inadequate blood supply to the placenta leading to hypoxic environment^9–11^. Therefore, although the cause of PE is yet clear, its origin is early and hence detection is feasible already at weeks 10–14^12,13^. In a recently published study, it was found that the administration of aspirin from 11 to 14 weeks of gestation resulted in a lower incidence of PE than that with placebo^14^. Furthermore, Rogerge *et al*. found that The effect of low-dose aspirin for the prevention of PE is optimal only when initiated before or at 16 weeks of gestation^15^. Henceforth, a reliable test that will detect an elevated risk for developing PE at an early stage of the pregnancy, before gestational week 16, is vital. Over the years, numerous studies have been attempted to define biomarkers and therapeutic targets for PE diagnosis and treatment. A number of maternal characteristics have been recognized as risk factors for developing PE, among them ethnicity, age, parity, multiple pregnancy, and a history of PE in earlier pregnancies^16–18^. Additionally, a few potential biochemical markers were found for predicting and diagnosing PE, including angiogenic/anti-angiogenic factors such as soluble fms-like tyrosine kinase 1 (sFlt-1), placental growth factor (PlGF), and vascular endothelial growth factor (VEGF)^19–21^. Other potential biochemical biomarkers include placental proteins, free fetal hemoglobin (HbF), and kidney markers^16^. Furthermore, some potential mRNA markers for PE were found in maternal plasma, among them antiangiogenic genes (such as FLT1 and endoglin), hypoxia-inducible factors (such as hif1α) and corticotropin-releasing hormone^22–24^. Currently, no such markers was proven consistently, or have been implemented in clinical practice, consequently new and better biomarkers for PE early prediction are most needed.

Small non-coding RNAs (ncRNAs) are a diverse family of untranslated RNA molecules (<200 nucleotides) that is part of the transcribed genomic output^25,26^. The most common known small ncRNAs are microRNAs (miRNAs), which are ~22 nucleotides long RNA molecules that regulate gene expression by facilitating mRNA degradation or by inhibiting protein translation^27^. Other small ncRNAs include: Small nucleolar RNA (snoRNA), which modulate the biogenesis and activity of ribosomes by post-transcriptional modifications of ribosomal RNA (rRNA)^28,29^; Small nuclear RNA (snRNA), which facilitates mRNA splicing and regulate transcription initiation^30,31^; and Transfer RNA (tRNA) - the most abundant small ncRNA - which play a role in translation^32^. The role of small ncRNAs in human diseases has been investigated mainly in the context of gene expression regulation via miRNAs, and has been well studied in several conditions including neurogenesis^33^, diabetes^34^, and especially cancer^35–38^. Nevertheless, recent studies have shown that dysregulation of many other types of small ncRNAs, besides miRNAs, have functional relevance in cancer as well as in most human diseases, from neurological disorders to cardiovascular problems^39,40^. These emerging studies strengthen the assumption that small ncRNAs have a much larger role in human disease etiology than previously understood. In PE, just as in other diseases, miRNAs have been the first ncRNAs for which involvement has been investigated. Current accepted theory claims that PE begins with abnormal placentation that leads to a maternal inflammatory response^41^. MiRNAs have important roles in both the placentation process^42^, and in the regulation of uterine inflammatory response^43,44^. MiRNAs are also related to many other mechanisms associated to PE pathogenesis including angiogenesis^45–48^, hypoxia^49,50^, regulation of blood pressure^51^ and cell differentiation, apoptosis, and migration/remodeling^46,52–55^. Moreover, in recent years, abundant and differentially expressed miRNA species in placental samples from PE and control samples have been reported^46,56–59^, which opened up the possibility to find new effective markers for the diagnosis of PE^60^. Additionally, circulating miRNAs in maternal blood, which are associated with preeclamptic risk, were identified, thought they were found in in the second or third trimester, after the appearance of PE symptoms^61–66^. Currently no such markers have been found for an early diagnosis prior to the appearance of symptoms. Potential links between other small ncRNAs and PE are currently more tentative, thought several small ncRNAs have been associated with PE or with known mechanisms involved in PE pathogenesis. For example, misregulation of snoRNAs were recently found in placentas of women with severe PE^67^. Additionally, U1 snRNA was found to be related to inflammation by inducing inflammatory cytokine release^68^. Two tRNA types (tRNA^Val^ and tRNA^Gly^) were found to inhibit angiogenesis by modulating the function of endothelial cells^69^, and misregulation of tRNAs and snRNAs was detected in cells cultured under hypoxic conditions^70^. Overall, those and other accumulating evidences suggest that miRNAs as well as other small ncRNAs may have a role in PE development and can be potential biomarkers for PE early diagnosis. In the current study, we investigated the abundance of circulating small ncRNAs in the plasma of pregnant women in their first trimester, seeking transcripts that best separate the PE samples from those of healthy pregnant women, and thus can serve as potential biomarkers for preeclampsia early diagnosis.

## Results

We performed small ncRNAs Next Generation Sequencing of PE and control samples, in a nested case-control study, aiming to find ncRNAs that best separate PE and control samples, and to evaluate their predictive value for PE early diagnosis.

### Patient characteristics

RNA was extracted and small ncRNAs were sequenced from the plasma of 75 pregnant women: 35 women that developed early onset PE (i.e., PE that develops before 34 weeks of gestation), and 40 women with normotensive uncomplicated pregnancies (i.e., a control set). All women were at the end of their first trimester (gestational age 11^+0^–13^+6^ weeks). Importantly, various ethnicities were included in the study (see a summary of maternal characteristics in Table 1). All women at the control group were women without pre-existing medical conditions, with uncomplicated pregnancies resulting in a delivery of a phenotypically normal neonate at term and with normal birthweight for gestational age at delivery. None of these women had a history of chronic hypertension. Cases and controls were matched regarding maternal age, nulliparity, fetal gender, ethnicity and smoking status. They differed significantly for first trimester Mean arterial pressure (MAP) (*p*-value < 0.0001), Uterine artery pulsatility index (UT PI) (*p*-value < 0.0001), and chronic hypertension (*p*-value = 0.001), which are all known risk factors for PE development. Additionally, both groups are differed significantly for the existence of PE in a previous pregnancy which is a risk factor as well, nevertheless there is no association between the circulating ncRNA expression and previous PE. Perinatal outcome data, (i.e, gestational age at delivery and birth weight) was also significantly different between groups (*p*-value < 0.001), due to early- PE and control group definitions.

**Table 1:** Clinical characteristics of PE and control groups.

Characteristic	Control (n = 40)	PE (n = 35)	p-value
Maternal age, years (IQR)	31.3 (25.9–34.6)	29.9 (28.1–34.5)	0.9200
Body Mass Index, kg/m^2^ (IQR)	24.1 (22.6–28.7)	28.4 (24.4–31.7)	0.0125
Gestational age, weeks (IQR)	12.8 (12.3–13.2)	12.7 (12.2–13.1)	0.3916
Crown-rump length, mm (IQR)	63.9 (57.5–70.2)	62.9 (56.2–67.9)	0.3029
Mean arterial pressure (MAP), mm Hg (IQR)	83.2 (79.4–87.7)	97.1 (90.1–108.7)	<0.0001
Uterine artery pulsatility index (UT PI) (IQR)	1.5 (1.3–1.7)	2.5 (2–2.8)	<0.0001
Gestational age at delivery, weeks (IQR)	39.8 (39.4–40.7)	31.4 (29.4–33.2)	<0.0001
Birth weight, g (IQR)	3,420 (3,198–3,578)	1,222 (951–1,565)	<0.0001
Fetal Gender, n(%)			0.65
female	22 (55)	17 (42.5)	
male	18 (45)	18 (45)	
Ethnicity, n(%)			0.2848
Afro-Caribbean	15 (37.5)	19 (54.3)	
South Asian	1 (2.5)	1 (2.9)	
Caucasian	24 (60)	15 (42.9)	
Cigarette smokers, n(%)			0.11
No smoker	34 (85)	34 (97.1)	
Smoker	6 (15)	1 (2.9)	
Family history of preeclampsia, n(%)			0.41
Yes	2 (5)	4 (11.4)	
No	38 (95)	31 (88.6)	
Parity, n(%)			0.0018
Multiparous with no previous PE	15 (37.5)	6 (17.1)	
Multiparous with previous PE	0 (0)	8 (22.9)	
Nulliparous	25 (62.5)	21 (60)	
Chronic hypertension, n(%)			0.001
Yes	0 (0)	8 (22.9)	
No	40 (100)	27 (77.1)	

A comparison of maternal and pregnancy characteristics between the two groups: pregnant women that have developed preeclampsia (PE) and pregnant women with uncomplicated pregnancies (control). *P*-values were calculated using Fisher exact test for categorical variables, and using Mann-Whitney U test for continuous variables.

### Differential expression analysis

**Figure 1:**
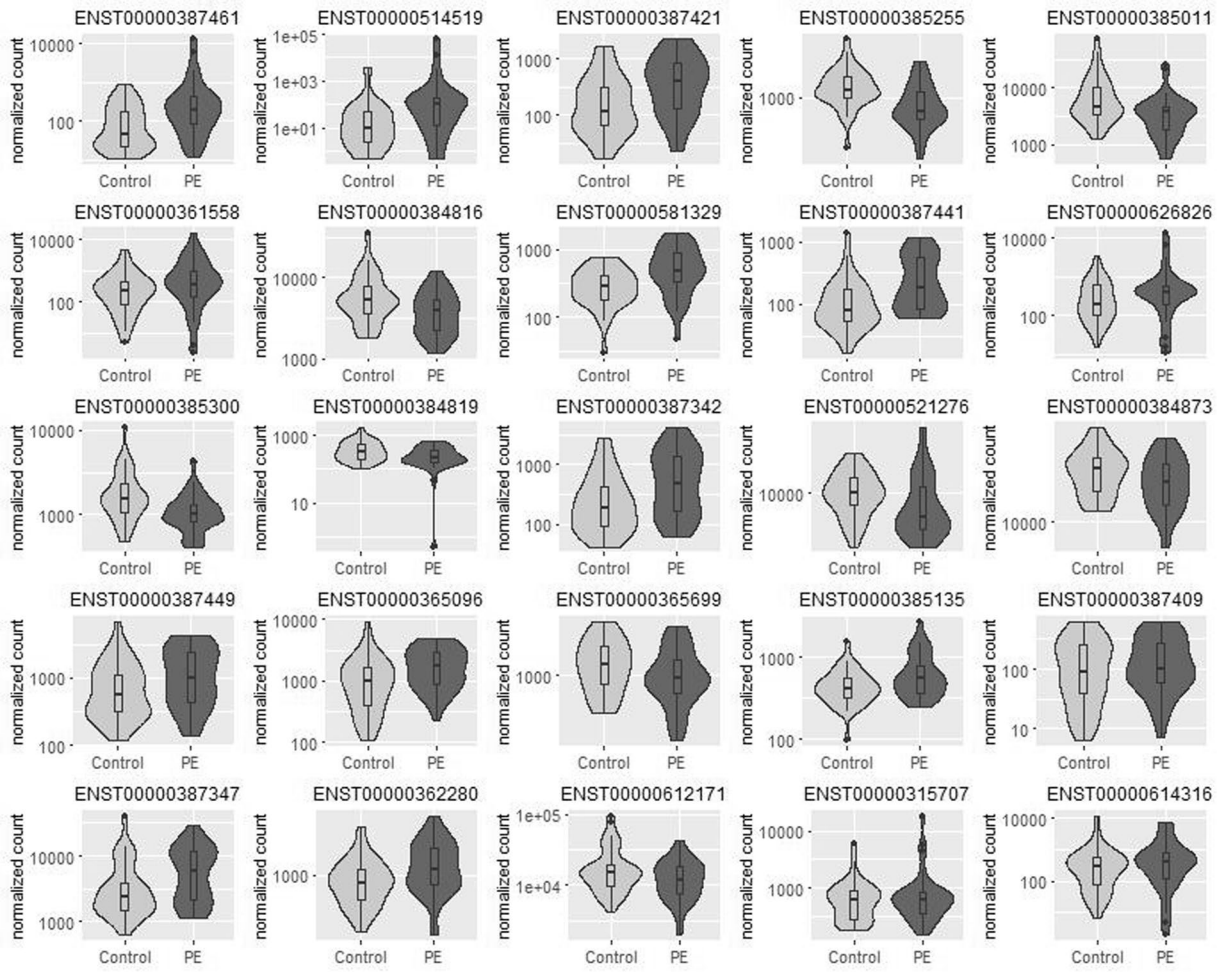
Normalized counts for differentially expressed transcripts in PE vs. control samples. Each of the most highly abundant transcripts in 35 PE vs. 40 control samples was tested for differential expression, and 25 transcripts were found to be differentially expressed (adjusted *p*-value < 0.05). Normalized counts are presented as violin and box plots. The upper and lower limits of the boxes represent the 75^th^ and 25^th^ percentiles. The upper and lower whiskers represent maximum and minimum values. The median is indicated by the line in each box. Outliers are indicated by circles.

Each of the most highly abundant transcripts in the women’s plasma was tested for differential expression in the PE vs. control samples, and 25 transcripts were found to be differentially expressed (adjusted *p*-value < 0.05, see Table 2 and Fig. 1): 16 transcripts were up-regulated, and 9 were down-regulated. Of these, 7 transcripts were tRNAs and rRNAs encoded in the mitochondria, 12 transcripts were microRNAs, 4 transcripts were long non-coding RNAs (linc), one transcripts was ribosomal RNA and one transcript was processed transcript (i.e., a non-coding transcript that does not belong to any of the categories in Ensembl database). We tested the correlation of each of the differentially expressed transcripts and the maternal clinical features, and observed moderate yet significant correlations between miR-4433b and 2 maternal clinical features: the uterine artery pulsatility index (UT PI; r = 0.395, adjusted *p*-value = 0.016), and the mean arterial pressure (MAP; r = 0.442, adjusted *p*-value = 0.003).

**Table 2:** Differentially expressed small ncRNAs in PE vs. control sample.

Transcript	Transcript ID	Transcript Biotype	Mean Counts	Fold Change	*P*-Value	Adjusted *p*-Value
mitochondrially encoded tRNA proline	ENST00000387461	Mitochondrial tRNA	490	4.25	1.65 × 10^−16^	1.57 × 10^−14^
mitochondrially encoded tRNA lysine	ENST00000387421	Mitochondrial tRNA	433	2.27	3.43 × 10^−6^	1.63 × 10^−4^
microRNA 182	ENST00000385255	miRNA	1,325	0.54	5.45 × 10^−6^	1.73 × 10^−4^
microRNA 10b	ENST00000385011	miRNA	7115	0.50	8.96 × 10^−6^	2.13 × 10^−4^
mucin 2, oligomeric mucus/gel−forming	ENST00000361558	processed transcript	901	2.34	1.68 × 10^−5^	3.19 × 10^−4^
microRNA 25	ENST00000384816	miRNA	5,585	0.61	5.38 × 10^−5^	6.39 × 10^−4^
RP11–259O2.3-001	ENST00000514519	lincRNA	409	2.97	4.92 × 10^−5^	6.39 × 10^−4^
microRNA 4433b	ENST00000581329	miRNA	473	1.71	4.98 × 10^−5^	6.39 × 10^−4^
mitochondrially encoded tRNA histidine	ENST00000387441	Mitochondrial tRNA	247	1.95	9.21 × 10^−5^	9.72 × 10^−4^
HELLP associated long non-coding RNA	ENST00000626826	macro lncRNA	729	2.02	1.08 × 10^−4^	1.03 × 10^−3^
microRNA 99b	ENST00000384819	miRNA	344	0.65	1.57 × 10^−4^	1.31 × 10^−3^
microRNA 143	ENST00000385300	miRNA	1,632	0.62	1.66 × 10^−4^	1.31 × 10^−3^
mitochondrially encoded tRNA valine	ENST00000387342	Mitochondrial tRNA	664	1.99	2.11 × 10^−4^	1.54 × 10^−3^
microRNA 151a	ENST00000521276	miRNA	10,021	0.75	5.68 × 10^−4^	3.85 × 10^−3^
microRNA 191	ENST00000384873	miRNA	31,187	0.75	6.26 × 10^−4^	3.97 × 10^−3^
RNA, 5.8 S ribosomal pseudogene 4	ENST00000365096	rRNA	1,652	1.68	1.65 × 10^−3^	9.21 × 10^−3^
mitochondrially encoded tRNA serine 2 (AGU/C)	ENST00000387449	Mitochondrial tRNA	1,250	1.72	1.61 × 10^−3^	9.21 × 10^−3^
microRNA 146b	ENST00000365699	miRNA	1,323	0.75	2.67 × 10^−3^	1.41 × 10^−2^
microRNA 221	ENST00000385135	miRNA	587	1.44	3.97 × 10^−3^	1.98 × 10^−2^
mitochondrially encoded tRNA tyrosine	ENST00000387409	Mitochondrial tRNA	173	1.53	4.41 × 10^−3^	2.09 × 10^−2^
mitochondrially encoded 16 S RNA	ENST00000387347	Mitochondrial tRNA	6,218	1.63	4.82 × 10^−3^	2.18 × 10^−2^
microRNA let-7g	ENST00000362280	miRNA	1,073	1.27	9.85 × 10^−3^	4.26 × 10^−2^
long intergenic non-protein coding RNA 324	ENST00000315707	lincRNA	1,087	1.50	1.03 × 10^−2^	4.27 × 10^−2^
AC113133.1-201 (microRNA-486)	ENST00000612171	miRNA	17,569	0.70	1.11 × 10^−2^	4.38 × 10^−2^
AC020956.3-001	ENST00000614316	lincRNA	902	1.71	1.18 × 10^−2^	4.47 × 10^−2^

Each of the most highly abundant transcripts in the women’s plasma was tested for differential expression in 35 PE vs. 40 control samples, and 25 transcripts were found to be differentially expressed (FDR adjusted *p*-value < 0.05).

In order to validate the microRNAs expression obtained by the small RNA sequencing, we examined five microRNAs expression using qPCR in 14 samples: 6 PE and 8 controls. We tested the correlation between the microRNAs expression obtained by sequencing and by qPCR in the relevant samples, and found a significant correlation in all tested microRNAs (see Supplementary Table S1), which confirms the microRNA counts obtained by sequencing.

To assess the 25 differentially expressed transcripts expression later in the pregnancy, after the appearance of PE symptoms, we sequenced small ncRNAs in the plasma of a subset of 40 women in weeks 20-22: 20 PE and 20 controls. As this set size is half of the original size and thus insufficiently powered, out of the 25 differentially expressed transcripts, only 9 transcripts were significantly differentially expressed in the limited set of 40 first trimester samples (adjusted *p-*value < 0.05, see Supplementary Table S2). In the second trimester, 4 transcripts out of the 25 were differentially expressed (adjusted *p-*value < 0.05, see Supplementary Table S3). Nonetheless, none of the transcripts displayed significant PE-dependent change in expression over the trimesters (PE/trimester interaction test, see Supplementary Table S4). These results suggest that fold changes observed in the first trimester are maintained during the second trimester as well, after the appearance of PE symptoms, though more samples from both gestational ages are required to further investigate this matter.

### Preeclampsia/Control Samples Classification

**Figure 2:**
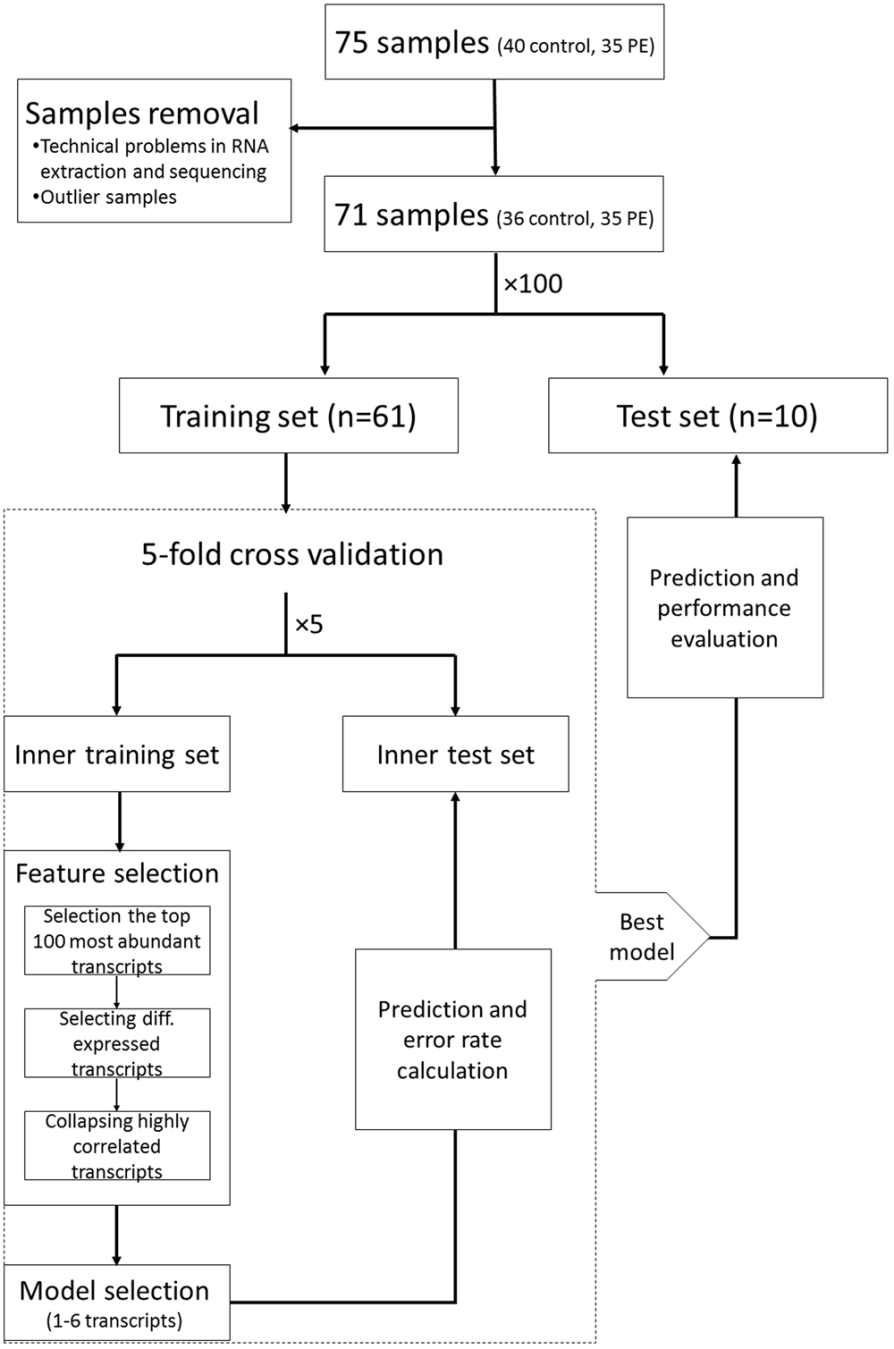
Schematic diagram of the workflow for PE/control samples classification. Data is randomly divided into a training set and a test set. 5-fold cross validation procedure is used on the training set to obtain a logistic regression model that best classifies training-set samples, and then it is tested on the blind test set. This process is repeated a 100 times, each time with a random partition to training and test set, in order to increase the stability and generalization of the results, and to estimate the goodness of the procedure on a new blind data set. The classification accuracy in the blind test set and related statistics are calculated in each of the iterations, and are summarized for overall evaluation of the pipeline.

**Figure 3:**
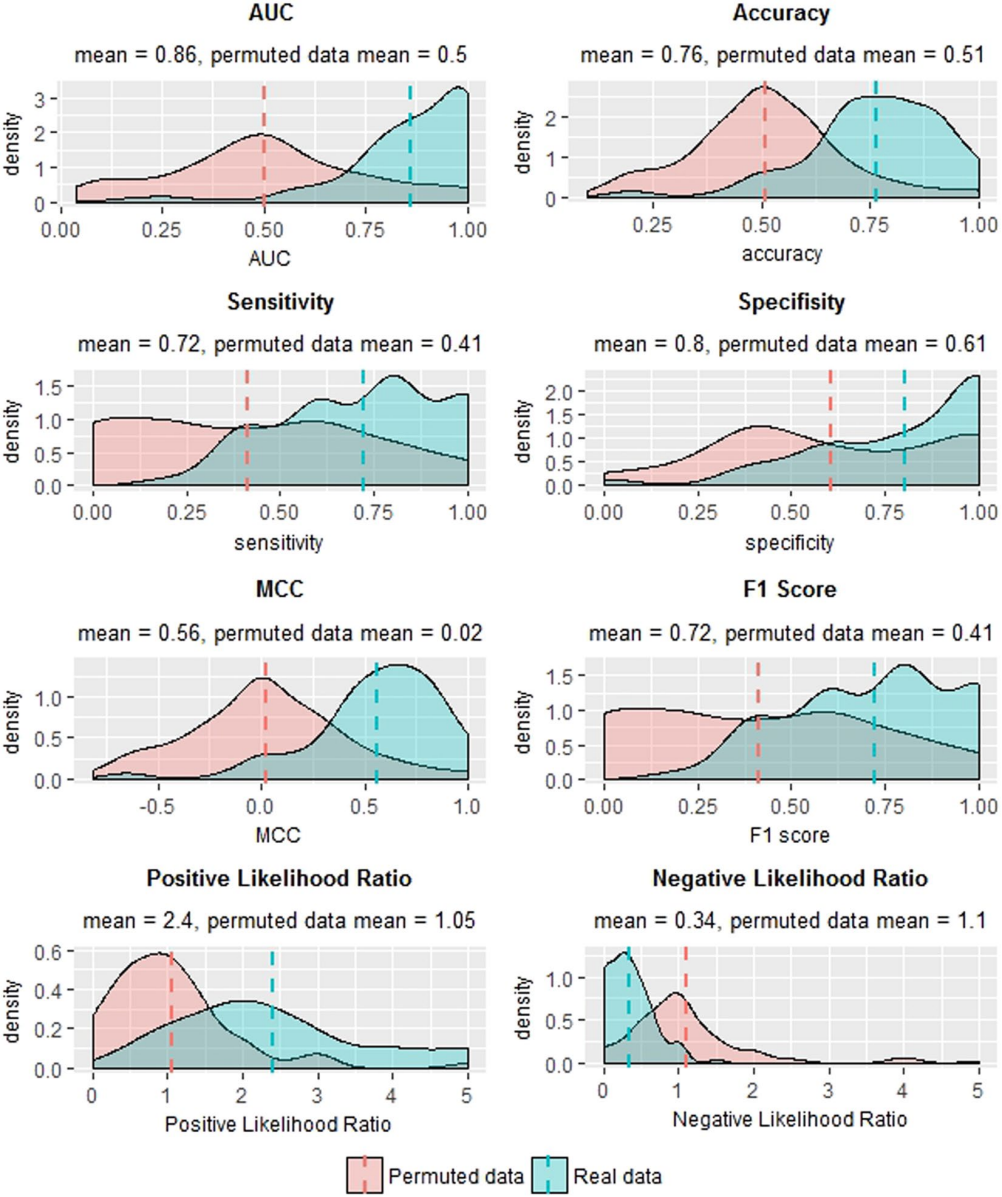
Classification results on real and permutated data sets. Density plots of statistical measures obtained by 100 iterations of our classification procedure on real (blue) and permutated (red) data sets. Real dataset included 35 PE and 40 control samples. Permutated dataset included the same samples after random shuffling of their conditions (i.e., PE/control). Means are indicated as well. Sensitivity: true positives out of all positives; Specificity: true negatives out of all negatives; Accuracy: true classifications out of all classifications; Matthews’s correlation coefficient (MCC): a correlation coefficient between the observed and predicted binary classifications; AUC: area under the ROC curve; F1 Score: the harmonic mean of precision and sensitivity; Positive Likelihood Ratio: sensitivity/(1-specificity); Negative Likelihood Ratio: (1-sensitivity)/specificity.

We proceeded to build a generalized pipeline for PE sample classification, and to estimate its performance. Since none of the differentially expressed transcripts can fully discriminate PE from control samples (see Fig. 1), we decided on utilizing a multivariable model that combines the differentiate ability of several transcripts in a synergetic manner. To this end, we chose to train a logistic regression model via a cross validation (CV) procedure and test it on blind test sets in an iterative process (see Fig. 2). In each cycle we randomly divided the samples dataset into a training set and a test set, and then trained a logistic regression model using a 5-fold cross validation procedure on the training set alone (see details in Materials and Methods). Briefly, the training set was divided into five non-overlapping and equally sized subsets, a logistic regression model was trained on four subsets and tested on the remaining subset. This process was repeated five times. The model features consisted solely of the ncRNAs expression; in each CV step a pre-processing feature selection was conducted, that included differential expression analysis only on the training set. Additionally, in each CV step we performed exhaustive search over all possible models. We limited the models’ size to a maximum of six transcripts, in order to avoid over fitting of the data and due to future practical clinical use. The selected model was then applied on the reaming subset in the CV step and error rate was calculated. The model that obtained the lowest error rate in all five CV steps, was then applied to the blind test set and its performance was evaluated. We repeated this procedure 100 times, each time with a random partition to training and test sets, in order to increase the stability and generalization of the results, and to estimate the goodness of the procedure on a new blind data set. Using this procedure we achieved a mean AUC of 0.86 (SE = 0.02) and accuracy of 0.76 (SE = 0.02). The mean sensitivity at false positive rates of 10% and 5% were 0.45 (SE = 0.017) and 0.28 (SE = 0.016) respectively. Figure 3 displays a summary of statistical measures calculated for this procedure. For the purpose of validating the computational pipeline, we conducted permutation tests to the classification process. We randomly permutated the samples’ conditions (i.e., PE/control) and followed the steps as described above. As shown in Fig. 3, the classification statistics in the permutation tests were distributed normally and followed the results of a random classifier, which further strengths our method’s effectiveness, and demonstrates the predictive value of circulating ncRNA.

## Discussion

PE is a pregnancy-associated multi-system disorder appearing at the second half of pregnancy, and is the leading cause of maternal and perinatal mortality and morbidity. The cause of PE is unknown, though it involves inadequate blood supply to the placenta leading to hypoxic environment^9–11^. PE remains unpredictable and is diagnosed only in the second or third trimester, consequently there is an increasing demand for better understanding of PE and development of an early diagnostics test. Although clinical symptoms are late, PE starts with placental dysfunction in the first trimester, hence early detection is feasible as early as weeks 10–14^12^. Developing a non-invasive efficient screening procedure to identify women at risk of PE would be beneficial for early targeted preventive/prophylactic interventions. Recently reported meta-analysis suggests that the effect of low-dose aspirin for the prevention of PE is optimal only when initiated before or at 16 weeks of gestation, hence women at high risk for PE should be identified in early pregnancy^15^. Some potential biochemical and mRNA markers for PE early prediction were previously reported^16^, though none has been proven to effectively predict PE. Small ncRNAs have important roles in many cellular processes such as gene regulation, translation, splicing, and many more. They can be purified from the plasma of first trimester pregnant women in sufficient amounts for accurate identification and quantification, which suggest their potential value as biomarkers for PE non-invasive early diagnosis. Though circulating miRNAs in maternal blood that are associated with preeclamptic risk were identified in later stages of the pregnancy^61–66^, currently no such markers were found for an early diagnosis prior to symptoms appearance.

Using small RNA sequencing analyses, we identified significant changes in the circulating ncRNA abundance in maternal plasma samples of first trimester PE pregnancies, compared with uncomplicated pregnancies. Seven of the up-regulated transcripts (and none of the down-regulated ones) were tRNAs and rRNAS encoded in the mitochondria. A growing body of evidence suggests that mitochondrial dysfunction is manifested by oxidative stress, compromised differentiation, and invasion of trophoblasts, which have been associated with PE pathogenesis^71–76^. Moreover, Qiu *et al*. found that the odds of PE were positively correlated with the copy number of mitochondrial DNA in maternal blood^77^. Our results suggest that in addition to mitochondrial DNA, mitochondrial non-coding RNA might also be associated with the development of PE. To the best of our knowledge, this is the first evidence of elevated levels of mitochondrial ncRNAs in the maternal plasma of PE patients. Further prospective research is required to assess these results and to investigate the mechanisms through which altered mitochondrial RNA play a role in the pathogenesis of PE.

Furthermore, 12 out of the 25 differentially expressed transcripts were microRNAs, importantly most were previously related to known mechanisms in PE pathogenesis. For example, miR-10 was down-regulated in PE vs. control samples, in agreement with a previous study that reported down-regulation of miR-10 in preeclamptic placenta compared with normal placentas from uncomplicated pregnancies^59^. MiR-10 directly targets vascular endothelial growth factor receptor 1 (VEGF-R1, Flt-1) and its soluble splice variant, sFlt-1, both anti-angiogenic factors^78^. Hence, down-regulation of miR-10 causes increased expression of both sFlt-1 and Flt-1, and significantly impairs the angiogenic behavior of human endothelial cells^78^. It is well known that angiogenesis is a major mechanism involved in PE pathogenesis. Both sFlt-1 and Flt-1 bind vascular endothelial growth factor (VEGF) and placenta growth factor (PlGF), which play a key role in promoting angiogenesis. A number of studies found elevated sFlt-1 levels and reduced levels of free VEGF and PlGF prior to the onset of the clinical symptoms of PE in blood samples from pregnant women who later developed PE^19,79,80^, which support our results of reduced miR-10 expression.

Most of the remaining differentially expressed micoRNAs were also previously related to angiogenesis (miR-143^45^, miR-221^47,48^, and miR-182^46^), as well as to other mechanisms in PE pathogenesis such as inflammation (miR-221^81^), hypoxia (miR-99^49^ and miR-151a^50^), regulation of blood pressure (miR-143^51^) and cell differentiation, apoptosis, and migration/remodeling (miR-143^52^, miR-191^53^, miR-182^46^, miR-25^54^, and let-7 family^55^). Moreover, similarly to miR-10, altered expression of miR-143^82^, -221^83^, -182^46^, -25^84^, -151a^85–87^, and -191^84^ have been detected in placentas from preeclamptic pregnancies in previous studies. Two of the 12 circulating microRNAs: mir-182^88^ and miR-221^66^, were also shown as differentially abundant in PE plasma samples in the third trimester. Additionally, we observed moderate yet significant positive correlations between miR-4433b and 2 maternal clinical features, which may suggest prognostic value for it.

From the remaining differentially expressed transcripts, 4 were long non-coding RNAs (linc). Interestingly, HELLP associated long non-coding RNA (LINC-HELLP) was over-expressed in PE vs. control samples. HELLP syndrome is a pregnancy-associated disease, a severe variant of PE, inducing hemolysis, elevated liver enzymes, and low platelet levels in the mother. LINC-HELLP is a novel lincRNA that was recently identified^89^. It is localized in first-trimester extravillous trophoblasts and negatively affects the differentiation of the extravillous trophoblasts^90^. Mutations in LINC-HELLP identified in HELLP families negatively affected trophoblast differentiation^89^, all of which support our findings.

Due to PE heterogeneity and complexed nature, it is unlikely to be accurately early detected by a single variable. Indeed none of the differentially expressed ncRNAs displayed a perfect separation of PE and control samples, hence we utilized a multivariable model that incorporates several transcripts for PE classification. Based on the differentially expressed ncRNA expression, we built a classification pipeline for PE, and displayed its efficiency. Our pipeline generates a generalizable logistic regression model using a 5-fold cross validation on numerous random partitions into training and test sets. We chose a rather strict machine learning procedure for evaluating the classifier, by using multiple randomly chosen blind test sets, in addition to the cross validation method. This procedure enables us to estimate the accuracy of the classifier given a new unseen data set. Additionally, we trained our model on samples from several ethnicities in order to increase its generalization ability. We acknowledge that additional studies are essential in order to validate the differentially expressed ncRNAs, and to test the classification method. Those validation studies should include larger datasets with samples from several other ethnicities and countries. Our findings suggest the predictive value of circulating small ncRNAs in the first trimester, and demonstrate their application in classification PE and control samples. Further study is required in order to determine whether integrating to the model other clinical features could improve its performance.

Our study has several limitations, which are derived mainly from the relatively limited number of samples in our dataset. Using a small dataset to train and test a prediction model might lead to an overestimation of the performance. In order to reduce this effect, we applied a cross validation procedure in 100 repeats and tested the model on numerous outer random test sets. Nevertheless, we acknowledge our results might still be overestimated, and further validation on an independent dataset is required. Acquiring more samples of various origins and integrating more predictive clinical features, will enable to increase our model generalization, test its performance, and to further confirm the differentially expressed ncRNAs.

In summary, our study suggest the potential of circulating small ncRNA as detectible and accurate biomarkers, which should be further validated in additional studies. Our findings lay the foundation for producing a novel early non-invasive diagnostic tool for PE, which will serve as an effective intervention, and consequently, reduce the life-threatening risk for both the mother and fetus.

## Materials and Methods

### Samples Collection

This was a nested case-control study drawn from a large prospective screening for adverse obstetric outcomes in women who were attending for their routine first hospital visit in pregnancy at King’s College Hospital, UK. This visit, which was held at 11^+0^ to 13^+^6 weeks gestation, included (1) recording of maternal characteristics, medical history and clinical measurements (2) collection of blood samples. Gestational age was determined from the fetal crown-rump length^91^. The prospective study period was from October 2006 to January 2013. The cases of PE were selected at random, and each case was matched to controls that were sampled on the same or next day. This study consisted of 75 pregnant women: 35 women that developed early-PE (i.e., > 34 weeks of gestation) and 40 healthy women with uncomplicated pregnancies (i.e., control set). All control set women delivered at term a phenotypically normal neonate and with birth weight between the 5th and 95th percentiles for gestational age at delivery. None of these women had history of chronic hypertension. For a subset of 40 women, blood samples were taken also in the second trimester (weeks 20^+0^-23^+6^). The women gave written informed consent to participate in the study, which was approved by the NHS Research Ethics Committee. All methods were performed in accordance with the relevant guidelines and regulations. Data on pregnancy outcome were collected from the hospital maternity records or the general medical practitioners of the women. The obstetric records of all women with preexisting or pregnancy-associated hypertension were examined to determine whether the condition was preeclampsia, as defined by the International Society for the Study of Hypertension in Pregnancy^92^.

### Small ncRNA Extraction and Sequencing

RNA was extracted via miRNeasy Serum/Plasma Kit, and quantified using a NanoDrop spectrophotometer (ND-1000). The spectrophotometric absorbance parameters of the samples were: 260/280 nm ~1.8 and 260/230 nm ~1.8. Small RNA libraries were prepared for deep sequencing using Illumina’s TruSeq small RNA sample preparation kit. During this process, samples were ligated with 3′ and 5′ adapters, reverse-transcribed and then amplified using a PCR. Libraries of cDNA were prepared from 140–160 bp PCR products (representing 20–40 nt RNA molecules) and sequenced in separate lanes on an Illumina HiSeq 2500 instrument at the Technion High Throughput Sequencing Unit.

### Sequencing Reads Profiling and Differential Expression Analysis

Sequence reads were analyzed as follows:

1. fastq-mcf tool (http://code.google.com/p/ea-utils/wiki/FastqMcf) was used for adapter sequences clipping, low quality (i.e., quality 30) bases trimming and filtering out short reads (i.e., reads with less than 16 nt).
2. Reads were mapped against Ensembl database for human ncRNAs release 83^93^ using Burrows–Wheeler transform based alignment tool (BWA)^94^. Only uniquely mapped reads with up to 2 mismatches were considered.
3. Differential expression analysis: We first performed Principal component analysis (PCA) using *prcomp* method in R. A PCA plot (i.e., a plot that shows the samples in the two-dimensional plane spanned by their first two principal components) was used to discover unwanted variation present in the data (i.e., batch effects), and to detect outlier samples. This analysis indicated the existence of two outlier samples, which were removed from downstream analysis, and a batch effect that matched the dates of the samples processing and sequencing. Our general goal was to find potential biomarkers for early detection of PE, therefore we focused on transcripts that had substantial expression, and sought those who were differentially expressed. We applied DESeq2^95^ (in R) on the 100 most abundant transcripts to obtain a list of differential expressed transcripts between control and PE samples. We included batch variable in the DESeq2 design in order to correct for batch effect. Only transcripts with *p-*value < 0.05 after false discovery rate (FDR) adjustment were considered.
4. The differentially expressed transcripts expression in the limited set of 40 women was compared between the first and second trimesters using DESeq2^95^. We first performed differential expression analysis on the limited set in each trimester separately as explained above. We then combined first and second trimester samples and used a design formula that models the condition (i.e., PE/control) difference at the first trimester, the difference over trimesters, and any condition-specific differences over trimesters (i.e., an interaction term condition:trimester). We performed a likelihood ratio test with a reduced model which does not contain the interaction term, to test whether the condition induces a change in gene expression at the second trimester compare to the first trimester. Only transcripts with *p-*value* < *0.05 after false discovery rate (FDR) adjustment were considered.

### Sample Classification

Our goal was to build a generalized classifier and to estimate its performance. To this end we chose to use logistic regression in a cross validation (CV) procedure. In order to obtain more generalizable models, the cross validation concept was applied 100 times. In each cycle we divided the sample into training and test sets, and applied 5-fold CV on the training set. I.e., the training set was divided into 5 non-overlapping and equally sized subsets, a logistic regression model was trained on 4 subsets and tested on the remaining subset. This process was repeated 5 times, thus all subsets were used as a test set in each step. We have applied a feature selection procedure in each CV cycle and narrowed down the list of transcripts to differentially expressed transcripts that have substantial expression in the current subset (i.e., in the top 100 most abundant transcripts). We then collapsed highly correlated transcripts (i.e., Pearson correlation >0.7) and counts were normalized using DESeq2^95^. Batch effect was removed using *ComBat* method from SVA package in R^96,97^, that adjusts for known batches using an empirical Bayesian framework. For model selection we have used *glmulti* package in R^98^, that performs an exhaustive search over all possible models, fit each model to the current set using *glm* and ranks them by Akaike information criterion (AIC). The model was then applied on the reaming subset (i.e., the inner test set) and error rate was calculated. The model that obtained the lowest error rate in all CV steps, was selected in each iteration and was tested on the outer test set. After 100 iterations we have calculated the average sensitivity, specificity, area under the receiver operating characteristic curve (AUC) and related statistics. All performance statistics were calculated from prediction results on the outer test set in the 100 iterations. Sensitivities at 10% and 5% false positive rates were calculated using a 5-fold cross validation over all samples and averaged over 100 iterations.

### Quantitative polymerase chain reaction (qPCR) Validations

RT-qPCR was performed to validate the differentially expressed miRNAs obtained by small RNA-Seq using PE and control samples. RNA was extracted via miRNeasy Serum/Plasma Kit. The Applied Biosystems™ TaqMan™ Advanced miRNA Assay (Applied Biosystems; Thermo Fisher Scientific, Inc.) was used to test miRNA expression. PCR amplification and reading were performed with the StepOne™ Real-Time PCR System (Life Technologies; Thermo Fisher Scientific, Inc.). Expression values were calculated using the comparative threshold cycle method^99^, and normalized with cDNA concentrations. We used cDNA concentration as normalization factor since initial RNA concentrations were undetectable using either NanoDrop nor Qubit systems, and due to the absence of a priori known stable normalizer miRNA. The cDNA concentrations were measured using the QuBit dsDNA quantification system and high sensitivity assay reagents (Invitrogen). Pearson’s product moment correlation coefficient were calculated using *cor.test* method in R between sequencing normalized counts and qPCR normalized values (2^−Ct^). Outlier samples were removed from correlation calculation due to problems in RNA extraction for sequencing (one sample) or due to anomalous results in RT-qPCR (2 samples).

### Data availability

The datasets generated during and/or analyzed during the current study are available from the corresponding author on reasonable request.

## Electronic supplementary material

Supplementary Information
