# Supplementary material for: Early Detection of Preeclampsia Using Circulating Small non-coding RNA
Source: Sci Rep. 2018 Feb 21;8:3401. doi: 10.1038/s41598-018-21604-6 (PMC5821867; doi:10.1038/s41598-018-21604-6)
Supplement: Supplementary file 1 — Supplementary Information [file 41598_2018_21604_MOESM1_ESM.pdf]

Early Detection of Preeclampsia Using Circulating Small non-coding RNA

Liron Yoffe[1], Avital Gilam[1], Orly Yaron[1], Avital Polsky[1], Luba Farberov[1], Argyro Syngelaki[2], Kypros Nicolaides[2], Moshe Hod[1,3] and Noam Shomron[1]*

[1] Faculty of Medicine, Tel Aviv University, Tel Aviv, Israel

[2] Harris Birthright Research Centre for Fetal Medicine, King's College, London, UK.

[3] Rabin Medical Center, Petah-Tikva, Israel

* Corresponding author: nshomron@post.tau.ac.il, 972-3-6406594

Supplementary Table S1

miRNA	Fold Change (small RNA-Seq)	*p*-value	Correlation between sequencing and qPCR (r^2)	*p*-value
**microRNA 99b**	0.65	1.57E-04	0.83	0.017
**microRNA 151a**	0.75	5.68E-04	0.73	0.011
**microRNA 191**	0.75	6.26E-04	0.7	0.016
**microRNA 146b**	0.75	2.67E-03	0.85	0.00099
**microRNA 221**	1.44	3.97E-03	0.83	0.0015

Table 1 Correlation values between five differentially expressed miRNAs in PE vs. control samples and RT-qPCR experiments. Pearson's product moment correlation coefficient were calculated between normalized counts obtained by sequencing and qPCR normalized values (2^{-Ct}).

Supplementary Table S2

Transcript ID	Transcript Description	Transcript Biotype	Base Mean	Fold Change	P-Value	Adjusted P-Value
**ENST00000514519**	RP11-259O2.3-001	lincRNA	134.59	3.83	4.47E-05	0.0011
**ENST00000361558**	mucin 2, oligomeric mucus/gel-forming	processed_transcript	629.80	2.15	0.0005	0.0057
**ENST00000387461**	mitochondrially encoded tRNA proline	Mt_tRNA	307.32	2.43	0.0011	0.0069
**ENST00000385011**	microRNA 10b	miRNA	2,008.14	0.42	0.0011	0.0069
**ENST00000614316**	AC020956.3-001	lincRNA	481.48	1.97	0.0020	0.01
**ENST00000385255**	microRNA 182	miRNA	631.14	0.40	0.0026	0.01
**ENST00000612171**	AC113133.1-201	miRNA	10,042.65	0.43	0.0045	0.02
**ENST00000365699**	microRNA 146b	miRNA	445.87	0.48	0.0049	0.02
**ENST00000387347**	mitochondrially encoded 16S RNA	Mt_rRNA	4,147.66	0.48	0.02	0.04

Table 2 Significantly differentially expressed transcripts in PE vs. control samples in the limited data set of 40 first trimester samples, that were also differentially expressed in the analysis of the entire dataset.

Supplementary Table S3

Transcript ID	Transcript Description	Transcript Biotype	Base Mean	Fold Change	P-Value	Adjusted P-Value
**ENST00000385135**	microRNA 221	miRNA	371.00	5.40	1.09E-05	0.0003
**ENST00000514519**	RP11-259O2.3-001	lincRNA	309.82	5.08	2.77E-05	0.0003
**ENST00000387342**	mitochondrially encoded tRNA valine	Mt_tRNA	2,947.79	0.24	0.0014	0.0113
**ENST00000387421**	mitochondrially encoded tRNA lysine	Mt_tRNA	1,729.23	0.26	0.0022	0.0136

Table 3 Significantly differentially expressed transcripts in PE vs. control samples in the limited data set of 40 second trimester samples, that were also differentially expressed in the analysis of the entire dataset of first trimester samples.

Supplementary Table S4

Transcript ID	Transcript Description	Transcript Biotype	Base Mean	Fold Change	P-Value	Adjusted P-Value
**ENST00000521276**	microRNA 151a	miRNA	4,514.08	0.27	0.04	0.40
**ENST00000384873**	microRNA 191	miRNA	12,546.19	0.28	0.06	0.40
**ENST00000384819**	microRNA 99b	miRNA	129.85	0.31	0.07	0.40
**ENST00000362280**	microRNA let-7g	miRNA	653.84	0.36	0.08	0.40
**ENST00000387342**	mitochondrially encoded tRNA valine	Mt_tRNA	1,517.16	0.31	0.09	0.40
**ENST00000385135**	microRNA 221	miRNA	446.48	0.36	0.10	0.40
**ENST00000626826**	HELLP associated long non-coding RNA	macro_lncRNA	497.67	1.30	0.14	0.49
**ENST00000365699**	microRNA 146b	miRNA	413.93	0.42	0.17	0.52
**ENST00000385300**	microRNA 143	miRNA	861.77	0.45	0.19	0.52
**ENST00000385255**	microRNA 182	miRNA	617.00	0.47	0.23	0.52
**ENST00000612171**	AC113133.1-201	miRNA	10,420.59	0.48	0.24	0.52
**ENST00000387461**	mitochondrially encoded tRNA proline	Mt_tRNA	315.89	1.74	0.25	0.52
**ENST00000385011**	microRNA 10b	miRNA	2,488.02	0.53	0.27	0.53
**ENST00000514519**	RP11-259O2.3-001	lincRNA	209.70	0.60	0.39	0.63
**ENST00000384816**	microRNA 25	miRNA	2,728.09	0.59	0.40	0.63
**ENST00000387441**	mitochondrially encoded tRNA histidine	Mt_tRNA	356.59	1.70	0.40	0.63
**ENST00000387449**	mitochondrially encoded tRNA serine 2 (AGU/C)	Mt_tRNA	1,106.02	1.44	0.52	0.74
**ENST00000614316**	AC020956.3-001	lincRNA	568.39	0.80	0.54	0.74
**ENST00000315707**	long intergenic non-protein coding RNA 324	lincRNA	651.28	0.83	0.57	0.74
**ENST00000387421**	mitochondrially encoded tRNA lysine	Mt_tRNA	926.67	0.71	0.60	0.74
**ENST00000387409**	mitochondrially encoded tRNA tyrosine	Mt_tRNA	207.66	1.27	0.64	0.74
**ENST00000387347**	mitochondrially encoded 16S RNA	Mt_rRNA	6,306.61	1.28	0.68	0.74
**ENST00000365096**	RNA, 5.8S ribosomal pseudogene 4	rRNA	1,782.07	0.81	0.69	0.74
**ENST00000581329**	microRNA 4433b	miRNA	200.30	0.79	0.72	0.75
**ENST00000361558**	mucin 2, oligomeric mucus/gel-forming	processed_transcript	792.728	0.94	0.87	0.87

Table 4 Differentially expressed transcripts in PE vs. control samples obtained by analysis of the entire first trimester data set and their fold cahnges difference between the first and second trimester. Fold changes are a ratio of first trimester fold change divided by second trimester fold change.
